# Modifying Bitumen with Recycled PET Plastics to Enhance Its Water Resistance and Strength Characteristics

**DOI:** 10.3390/polym16233300

**Published:** 2024-11-26

**Authors:** Assel Jexembayeva, Marat Konkanov, Lyazat Aruova, Akpan Kirgizbayev, Lailya Zhaksylykova

**Affiliations:** 1 Department of Industrial and Civil Construction Technology, L.N. Gumilyov Eurasian National University, Astana 010008, Kazakhstan; asseljexembayeva@onmail.com (A.J.); lyazaruova@outlook.com (L.A.); akp_kirgizbayev@outlook.com (A.K.); leizhaksylykova56@outlook.com (L.Z.); 2 ENU Lab, L.N. Gumilyov Eurasian National University, Astana 010008, Kazakhstan

**Keywords:** bitumen, polyethylene terephthalate, strength, modification, stress, mechanical properties, polymer

## Abstract

This study investigates the modification of bituminous mixtures by varying percentages of PET particles (1%, 3%, 5%, 8%, 10%, and 12% PET). The following methods were employed to analyze the samples: the ring-and-ball softening point determination method (ASTM D36/D36M-14), the Fraass breaking point determination method (EN 12593: 2015), the elongation determination method (EN 13589: 2014), and the needle penetration depth determination method (EN 1426: 2015). Optimal bitumen/PET ratios were identified to obtain modified bituminous mixtures (MBMs) with enhanced operational characteristics (5% and 8% PET). The physical and mechanical properties of the investigated bitumen samples and PET were determined. A comparative analysis of the modified bituminous mixture samples based on their physical and mechanical properties was conducted. Microstructures of the surface of modified bituminous mixture samples with varying modifier contents were obtained. An X-ray structural analysis was performed on the samples of modified bituminous mixtures with varying PET contents. The dependencies of the moisture absorption rate on time were determined for the samples of modified bituminous mixtures with different modifier contents. The values of the stress intensity factor were determined based on the number of loading cycles in fatigue tests using three-point bending for the samples of modified bituminous mixtures with varying modifier contents.

## 1. Introduction

In the context of chemical industry development, particularly in the production of polymer materials (plastics), and with the increasing global population, there is a consequential rise in the volume of plastic waste [1]. Research indicates that over the past decade, the global production of composite [2] and polymer materials [3] has sharply increased, with plastic consumption rising by approximately 50% [4]. However, around 80% of plastic does not biodegrade in nature and is instead discarded as waste in the environment [5].

This situation leads to a significant deterioration of the ecological environment, adversely affecting all living organisms in the ecosystem through the chemical leaching of plastics into soil and water [6]. Furthermore, landfills designated for waste storage occupy vast areas and contribute significantly to the increased cost of waste disposal [7].

Kazakhstan’s state policy in waste management has adopted a trajectory towards developing a “green” economy, prompting rapid advancement in the country’s secondary plastic recycling and the implementation of closed-loop material principles.

The primary sources of plastic waste are plastic bags and plastic bottles [8]. Approximately 15% of these wastes consist of polyethylene terephthalate (PET) residues—a thermoplastic polymer from the complex polyester class, characterized by its transparent semi-crystalline thermoplastic ether with a long chain. PET is valued for its ease of handling, durability, strength, chemical stability, and low gas permeability. The recycling rates for this plastic waste remain around 29% [9]. PET distinguishes itself with its low production cost [10] and serves as the primary raw material for the production of disposable packaging and utensils [11]. It possesses a simple chemical structure, (C_10_H_8_O_4_)n, existing as a solid and colorless substance in the amorphous state and as a white substance in its crystalline state. PET is a common and effective polymer additive in modified bituminous mixtures (MBMs) [10]. However, its high melting temperature (approximately 260 °C) presents certain challenges in its application for road surface production [12].

Pure bitumen exhibits high thermal sensitivity, softening at high temperatures and becoming brittle with cracking at low temperatures under loads. Additionally, pure bitumen is characterized by low mechanical properties and susceptibility to aging [13]. In order to optimize bitumen properties, it is modified with various additives, leading to an extended service life and improved resistance to tension and shear [14]. The key characteristics of modified bituminous mixtures include enhanced thermal sensitivity, aging resistance, elastic–plastic range, load and fatigue resistance, greater elasticity, and plasticity, among others [15]. Even a small addition of PET (2–10%) effectively influences the deformational characteristics of bitumen [16].

Thus, the studies on the application characteristics of recycled PET presented in this work are promising both for the road construction industry and for environmental considerations.

### 1.1. Literature Review

Road pavements are typically categorized into two types: flexible and rigid. Flexible road pavements are topped with bituminous coatings, whereas rigid road pavements (which are stiffer than flexible ones) have asphalt concrete layers on top. Flexible road pavements are laid in layers, with the maximum load bearing on the upper layers primarily being composed of high-quality materials, mainly bitumen [17].

Statistics from scientific studies on various compositions of bituminous binders indicate that the durability of road pavements with unmodified bitumen layers has decreased from the range of 6–8 years to approximately 3–5 years in modern road construction. Under conditions of intense traffic and extreme factors, traditional bituminous binders typically do not meet durability requirements [18].

Roads constructed from bitumen become brittle over time in cold temperatures, leading to cracking and destruction under loads. This results in the formation of cracks, which quickly expand and develop into potholes. Bituminous roads are also characterized by instability to temperature fluctuations, which can cause rapid wear of road surfaces [19].

The main types of damage caused to road pavements due to bitumen aging include washout, rutting, fatigue damage, damage from low temperatures, potholes, and multiple focal damages known as “alligator cracking” [12]. To minimize the occurrence of these defects, modified bituminous mixtures (MBMs) are produced, which possess enhanced physical and mechanical properties.

The production of bituminous mixtures is guided by economic feasibility and practicality, aiming to select the optimal ratio of aggregates and an adequate proportion of bitumen to ensure the required properties of the mixture are achieved, such as thermoplasticity, aging resistance, high mechanical properties, hydrophobicity, and more. Modified bituminous mixtures (MBMs) demonstrate superior performance characteristics in field conditions, are more economical, and contribute to environmental improvement by recycling plastic waste [20].

Temperature changes, moisture, mechanical loads, ultraviolet radiation, and other physico-chemical and mechanical impacts accelerate the aging process of bituminous mixtures [21]. Aging results in increased brittleness, degradation, and a loss of performance characteristics in bitumen [22]. To mitigate the negative effects of bitumen aging, scientific research employs modern methods for monitoring structure and properties [23]. Research shows that various chemical reactions, such as cracking, polymerization, and cross-linking, occur in bitumen under ultraviolet radiation, which reduces the longevity of the bituminous mixture [24]. To prevent these adverse effects, studies have investigated the impacts of various substances and nano-additives that slow down bitumen aging and enhance its resistance to damage [25]. Reference [14] highlights synthetic polymer modifiers that increase bitumen’s aging resistance, such as plastomers and thermoplastic elastomers. Among plastomers, the primary materials for bitumen modification include polypropylene (PP), high-density polyethylene (HDPE), polyethylene terephthalate (PET), ethylene butyl acrylate (EVA), and ethylene-vinyl acetate (EVA). Among thermoplastic elastomers, notable examples are styrene–isoprene–styrene rubber (SIS), butylene–styrene rubber (SBR), styrene–ethylene–butylene–styrene (SEBS), and styrene–butadiene–styrene rubber (SBS).

Many studies [22,24] focus on SBS and SIS copolymers as bitumen modifiers. These copolymers are the most common bituminous binders, forming a spatial polymer matrix at certain concentrations within the bitumen system. However, the cost of these modifiers is quite high. Consequently, research is being conducted to find more economically advantageous polymer modifiers, partially derived from the recycling of household waste, such as thermoplastic rubbers based on ethylene–propylene rubber (EPR), high-density polyethylene, and polyethylene terephthalate.

One of the most common and economically viable modifiers for the road construction industry is the synthetic material PET, derived from the recycling of raw oil. Incorporating PET into asphalt increases its rigidity and resistance to temperature fluctuations, thereby enhancing rutting resistance [11].

Scientific research on the use of PET as a modifying additive has demonstrated significant improvements in physical and mechanical properties, such as compressive strength [26], tensile strength [27], and flexural strength [28], as well as enhanced concrete plasticity, reduced bulk density in composites, and minimized solid waste issues [8].

Table 1 presents the characteristic features of modified bituminous mixture samples based on the analysis of certain surface microstructures. The images were obtained using fluorescence microscopy in [29].

The analysis of microstructures provides information on the compatibility of a specific modifier within the bitumen sample and the uniformity of phase distribution.

### 1.2. Problem Statement

The aim of this study is to investigate the performance properties of modified bitumen obtained through the chemical interaction between recycled PET and bituminous binder. For this purpose, it is essential to establish optimal ratios of bitumen and the modifying additive—PET waste—to achieve modified bituminous mixtures (MBMs) with enhanced performance characteristics.

To achieve the research objective, the following tasks must be addressed:

− Conduct a comparative analysis of modified bituminous mixture samples based on their physical and mechanical properties.
− Obtain the surface microstructures of modified bituminous mixture samples with varying modifier contents.
− Perform an X-ray structural analysis of modified bituminous mixture samples with different contents of PET additive.
− Determine the relationship between the moisture absorption rate and time for modified bituminous mixture samples with varying modifier contents.
− Identify the values of stress intensity factors as a function of the number of loading cycles during fatigue testing using three-point bending for modified bituminous mixture samples with different modifier contents.

## 2. Methods and Materials

The PET modifier was obtained through the mechanical recycling of plastic bottles. In the mechanical recycling process, the polymer mass was heated to 200 °C, extruded through holes to form filaments, and then cooled in a water tank. The resulting filaments were cut into pellets with sizes ranging from 3 to 5 mm, a molecular weight of 35,000, a melting temperature of 245 °C, and a glass transition temperature of 70 °C.

The effectiveness of bitumen modification was evaluated with PET waste additives in quantities of 1, 3, 5, 8, 10, and 12 wt%.

To obtain samples with the specified modifier content, the components were mixed in a high-speed colloid mill mixer for micro-cements. Based on the experimental data, the following conditions were selected:

− Temperature: 180 °C;
− Mixing time: 45 min;
− Mixing speed: 2000 rpm.

The process of obtaining bitumen modified with a plastic polymer began with mixing the mixture at a speed of 700 revolutions per minute for the first 15 min to facilitate the release of air bubbles from the resulting mixture and reduce air voids; after this, the mixer speed was increased to 2000 revolutions per minute, and mixing was continued for 30 min. These conditions were set for all formulations. After the mixing process, the material was allowed to cool down. Testing was conducted after 24 h.

The puncture of the bitumen mixture was performed by melting and cooling the sample under stringent conditions. Puncture depth was measured using a standard penetration device and needle. The penetration depth is defined as the distance in tenths of a millimeter that a standard needle vertically penetrates into the sample material at a constant temperature. Standard penetration testing was conducted at 25 °C for 5 s using an APN-360MG4 penetrometer based on bitumen and with polymer concentrations (1%, 3%, 5%, 8%, 10%, and 12%) by mass of bitumen.

The research methods used in this study are presented in Table 2.

In Table 3, the physical and mechanical characteristics of the investigated bitumen samples are presented. The density of the samples at 25 °C has an actual value of 1100 kg/m², which falls within the standardized range of 950–1500 kg/m² for bitumen. The needle penetration depth of the samples at 25 °C has an actual value of 96 mm, which also falls within the standardized range of 70–100 mm for bitumen.

The tensile elongation of the samples at 25 °C has an actual value of 54 cm compared to the standardized value of 62 cm for bitumen. At 0 °C, the tensile elongation of the samples has an actual value of 3.7 cm, which corresponds to the standardized value for bitumen. It is noteworthy that decreasing the test temperature by just 25 °C reduces the tensile elongation of the bitumen samples by a factor of 15.

The softening of the samples at 70 °C has an actual value of 68 °C, which falls within the standardized range of 0–70 °C for bitumen.

The brittleness of the samples at −15 °C has an actual value of −15 °C, which falls within the standardized range for bitumen from 0 to −15 °C. Thus, the conducted research on the physical and mechanical characteristics of the investigated bitumen samples has shown that their actual values align with the standardized values.

In Table 4, the physical and mechanical characteristics of the PET modifier samples are presented: molecular weight; density; tensile strength; elongation at break; longitudinal modulus of elasticity; impact viscosity; frost resistance; water absorption; decomposition temperature; melting temperature; and glass transition temperature.

To evaluate the chemical properties and microstructure of bituminous mixtures, studies were conducted using X-ray structural analysis and fluorescence microscopy. X-ray structural analysis of the samples of the modified bituminous mixture was performed at various percentages of PET additive: 1%, 3%, 5%, and 10%. The microstructure of the surface of the modified bituminous mixture sample (at 10× magnification) was determined for different percentages of the PET additive: 1%, 3%, 5%, and 8%. The thermograms of the bitumen, PET modifier, and modified bituminous mixture with 10% PET additive were generated using differential scanning calorimetry. This method involved recording the thermal effects occurring in the sample during heating at a constant rate and comparing them with a reference (an empty crucible). The water absorption of the samples (containing 1%, 5%, and 12% PET modifier) was determined by analyzing the mass gain curves of the samples on the substrate, followed by determining the rate of water absorption. The values of stress intensity factors as a function of the number of loading cycles during fatigue testing were established through three-point bending of the modified bituminous mixture samples (in beam form) with the following contents: 1% bitumen mixture modifier, 3% bitumen mixture modifier, and 5% bitumen mixture modifier.

## 3. Results

In Table 5, the results of assessing the effectiveness of bitumen modification with PET waste at 1, 3, 5, 8, 10, and 12% by mass are presented. A comparative analysis of the samples of modified bituminous mixtures and their physico-mechanical properties is shown, including the following: density (increases with an increasing percentage content of the modifier); needle penetration depth at 25 °C (decreases with an increasing percentage content of the modifier); elongation at 25 °C (shows optimal values at modifier percentages in the range of 5–10%); softening point (increases with an increasing percentage content of the modifier); brittle point temperature (decreases with an increasing percentage content of the modifier); water absorption (decreases with an increasing percentage content of the modifier); and tensile strength (increases with the PET content, showing optimal values at 5% and 8% modifier contents).

The comparative analysis of the physical and mechanical properties of the samples of modified bituminous mixes revealed that the modification of bitumen with PET particles increases the density, tensile strength, softening point, and tensile strength at the break while reducing the needle penetration depth and fracture temperature. The addition of PET leads to a decrease in the needle penetration depth and softening point, thereby enhancing the strength and elastic properties of the modified binder, and reducing susceptibility to deformation, rutting, and crack formation. This phenomenon may be attributed to the swelling of PET particles and their improved compatibility with bitumen.

With an increase in the amount of modifier, an increase in the tensile strength is observed, but only up to certain values (up to 8% PET). At 10% PET, the tensile strength decreases. This could be due to an excessive amount of modifier that fails to blend properly with the asphaltene phase, as well as an increased specific weight and the settling of plastic particles within the composition. A similar trend is observed for the tensile strength at break values.

**Figure 1:**
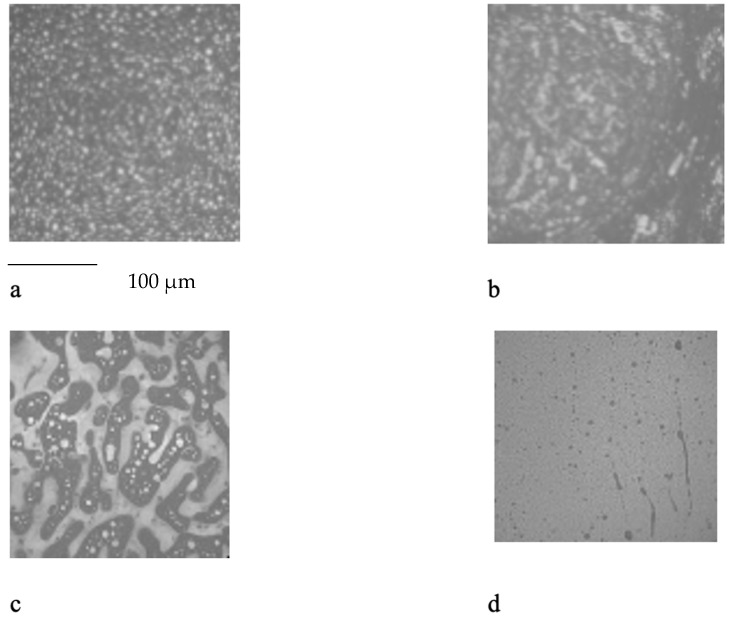
The microstructure of the surface of a sample of the modified bituminous mixture (10× magnification): (**a**) 1% modifier; (**b**) 3% modifier; (**c**) 5% modifier; and (**d**) 8% modifier.

The analysis of the data indicated that the PET plastic contents of 5% and 8% provide the best properties for modified bitumen and are considered optimal. Increasing the content of plastic is not advisable due to the increased production costs of modified bitumen and the reduction in some performance indicators. The results of the microstructure analysis of the samples of modified bituminous mixes with different contents of PET particles (1%, 3%, 5%, and 8%) are presented in Figure 1.

It has been established that the structure of the bituminous mixture varies with low and high polyethylene contents. At low concentrations (1% and 3%), individual particles of the polymer-enriched phase are visible in the microstructures, dispersed within the asphaltene-enriched phase. At a PET concentration of 5%, the structure is the most heterogeneous, with extensive areas of the polymer-enriched phase being observed. The inherent structural framework of the bitumen is replaced by a composite structure formed with the polymer consisting of both crystalline and amorphous components.

For samples of modified bituminous mixtures with an 8% PET particle content, spherical domains are characteristic, corresponding to the minimum phase boundary.

Thus, the obtained microstructures of the samples of modified bituminous mixtures with different percentages of PET particles correspond to typical microstructures of polymer-modified bituminous binders obtained in [29] and are shown in Table 1.

**Figure 2:**
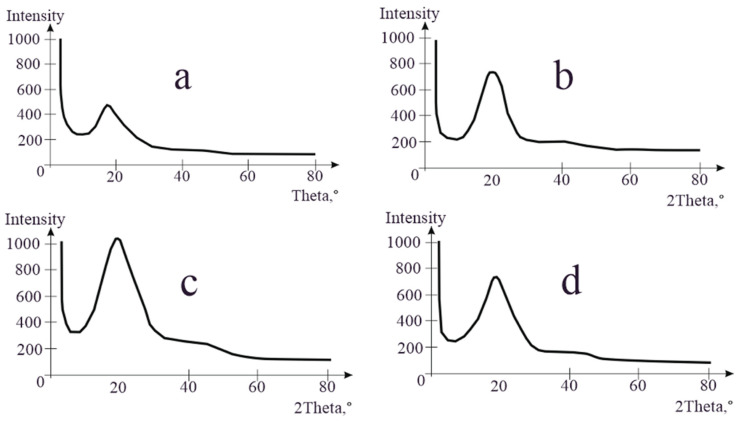
Results of X-ray structural analysis of samples of modified bituminous mixtures with different contents of PET additive: 1% (**a**); 3% (**b**); 5% (**c**); and 10% (**d**).

X-ray analysis studies were conducted to assess the chemical properties of the binders. Figure 2 presents the results of the X-ray structural analysis of the samples of modified bituminous mixtures with varying contents of PET additive: 1% (a); 3% (b); 5% (c); and 10% (d).

The obtained X-ray diffraction patterns show that the maximum peak intensity, approximately at 2*θ* = 20°, was observed in the bitumen sample with the minimal amount of PET. This value illustrates the characteristic property of bitumen, which consists predominantly of a uniform amorphous structure [34].

The intensity of X-ray peaks of the binders (especially at 5% PET) increased with an increasing amount of additives. When the modifier content reached ten percent, the intensity of the peak decreased. This can be attributed to the settling of some PET particles due to their higher density compared to bitumen.

**Figure 3:**
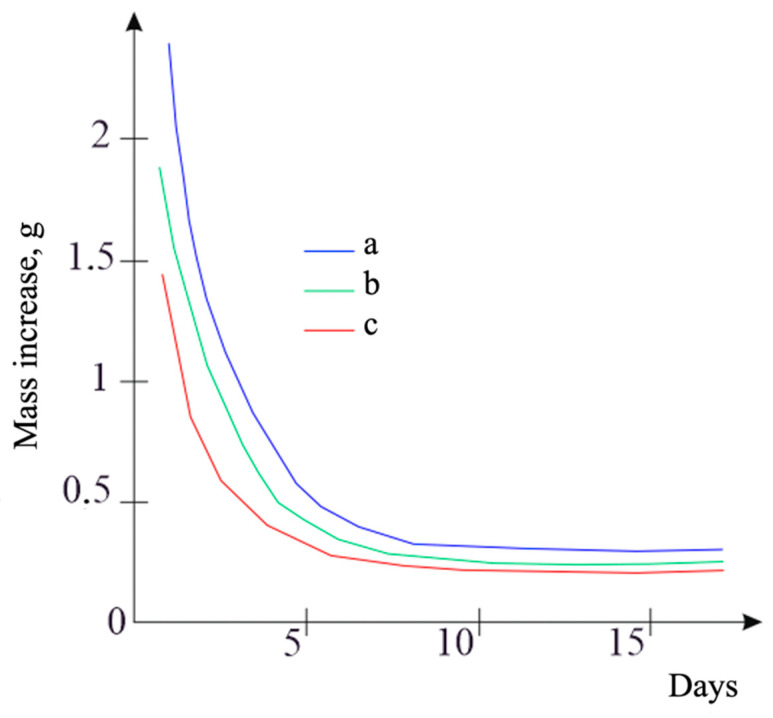
Dependence of moisture mass increase rate in sample over time for a—1% modifier in bituminous mixture; b—5% modifier in bituminous mixture; c—12% modifier in bituminous mixture.

To investigate the water resistance of the modified bituminous mixture samples, the dependencies of sample mass growth were constructed, and the rates of mass increase due to moisture were determined for various percentage contents of the modifier. Figure 3 presents the obtained dependencies of the moisture mass increase rate over time for the following: 12% modifier in the bituminous mixture, 5% modifier in the bituminous mixture, and 1% modifier in the bituminous mixture. It was determined that as the percentage content of the modifier increases, the mass of water absorbed by the sample decreases.

**Figure 4:**
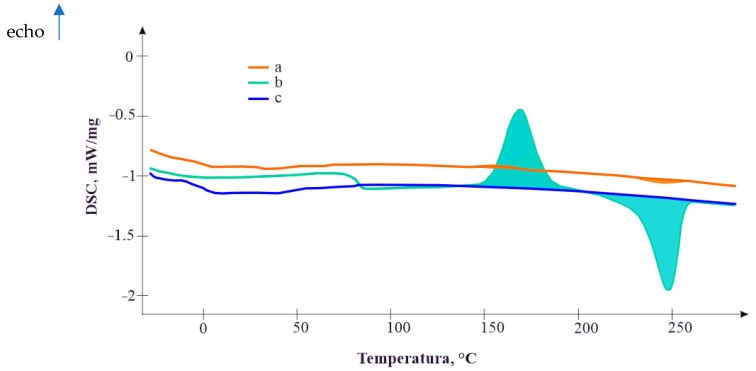
Thermograms of the physical and phase states of the PET modifier in bituminous mixture samples: a—modified bituminous mixture sample with 10% PET; b—PET modifier sample; c—bitumen sample.

Figure 4 depicts the thermogram of bitumen (curve a), the thermogram of the PET polymer (curve b), and the thermogram of bitumen with a ten percent addition of PET (curve c). The thermograms were obtained using differential scanning calorimetry. Samples of modified bituminous mixtures were heated at a constant rate with a recording of the thermal effects compared to an empty crucible, which served as a reference standard.

When using differential scanning calorimetry, the instrument’s software automatically determines changes in the phase composition of the sample based on variations in the Tg curves, which may not be visually discernible.

The conducted studies of thermal effects in the modified bituminous mixtures showed that bitumen does not exhibit phase transition at temperatures of up to 250 °C. The modifier, however, exhibits the following three phase transitions:

1. A glass transition onset at 77 °C;
2. A crystallization onset at 160 °C;
3. Melting within the range of 205–265 °C.

The analysis of the thermal effects of the modified bituminous mixtures revealed that the elasticity of the mixture decreases with the introduction of PET particles exceeding 20%, leading to increased cracking tendencies in the specimens. For pure polyethylene terephthalate (PET) without bitumen, the glass transition onset occurs at 82 °C, whereas in the presence of bitumen, it shifts to 71 °C. Similar observations were made for the melting temperatures, which decreased in the presence of bitumen compared to pure PET.

**Figure 5:**
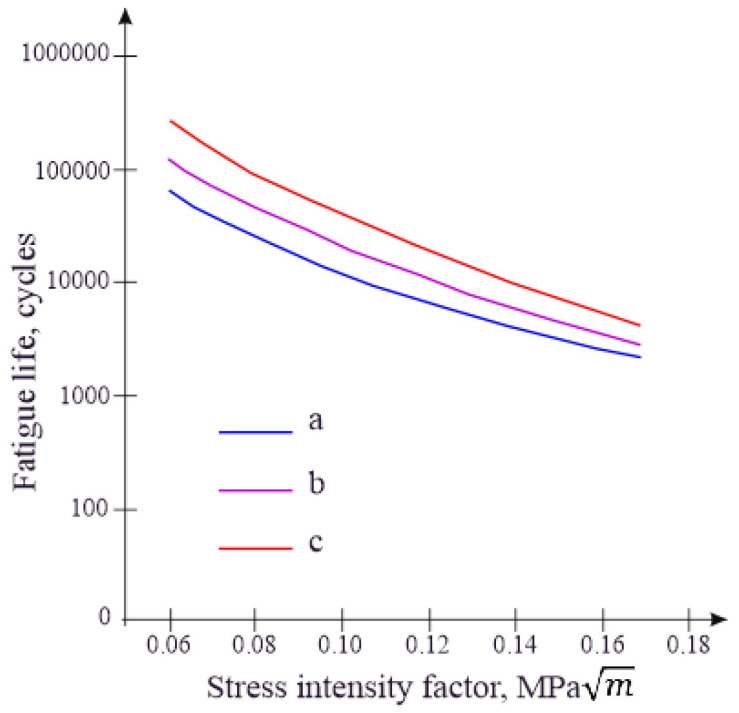
The stress intensity factor as a function of loading cycles in fatigue tests by three-point bending for modified bituminous mixtures with the following contents: (a) 1% modifier of bituminous mixture; (b) 3% modifier of bituminous mixture; and (c) 5% modifier of bituminous mixture.

Research was conducted to assess the impact of PET particles on the fatigue characteristics of the modified bituminous mixture. The values of the stress intensity factor as a function of loading cycles in fatigue tests using three-point bending are depicted in Figure 5.

The development of fatigue cracks is described by relationships based on the stress intensity factor at the crack tip [35]. The stress intensity factor characterizes a material’s resistance to crack propagation [36]. Therefore, to investigate the crack resistance of the samples of modified bituminous mixtures, relationships between their fracture viscosity and the number of cycles were constructed.

The results of the conducted research show that the fatigue durability of the mixture significantly increases after the addition of PET particles into the bituminous mixture, especially in low-stress zones, and with increasing stress, the differences between the samples at 1–3 decrease (Figure 5). Thus, the fatigue durability of the modified bituminous mixture can be significantly increased by adding a PET modifier, especially under low loads.

## 4. Discussion

In reference [37], as well as in our study, a detailed review was conducted on the application of PET additives to enhance the operational characteristics of bituminous mixtures. The literature survey in [37] demonstrates that polyethylene terephthalate additives improve the performance properties of road pavements: they reduce the penetration of bituminous mixtures and increase the softening temperature [38], plasticity [39], viscosity [40], tensile strength [41,42], density [43], fatigue strength [44], crack resistance [45], and resistance to residual deformation [46]. Similar results were obtained in our study, which is corroborated by research from [38,39,40,41,42,43,44,45,46].

Experimental work focused on enhancing the strength of flexible road pavements through the addition of plastic waste in various percentage ratios was conducted in [47]. The study concluded that to achieve satisfactory performance characteristics, 10% of bitumen in the bituminous layer can be replaced with plastic waste. In our research, we found that 10% PET enhances the characteristics of bituminous mixtures, but the optimal quantity is found to be at 5% and 8%.

The obtained results are consistent with those of other scientific studies available in open sources. For instance, references [48,49,50] demonstrated that the optimal amount of PET modifier in samples of polymer-modified bitumen is around 5–6% by mass. At this ratio, maximum values of viscosity, stiffness, and resistance to residual deformation are observed.

An X-ray structural analysis of bituminous mixture (MBM) samples revealed that using additives with low PET contents results in weak chemical bonding between bitumen and PET, but this bond strengthens with an increasing PET content, with the best outcome being observed with a 5% modifier. At higher PET ratios, PET no longer interacts with bitumen. The intensity of the peak decreases at 10% PET, while the optimal result is achieved with a 5% modifier. These findings are in line with reference [51], where an X-ray structural analysis of MBM samples identified the optimal PET additive ratio to be 6%.

Table 5 demonstrates that the softening point increases with the amount of PET. Similar results were obtained in reference [52]: the addition of PET to bitumen increases the softening point from 47 °C for regular bitumen to 94 °C for PET-modified bitumen.

Table 5 also shows that the density of modified bitumen increases with an increasing PET content, while the penetration depth at 25 °C decreases. Similar findings were reported in [52]: the specific gravity of regular bitumen increased with the addition of PET in amounts of 3, 5, 7, 9, 11, 13, 15, and 17%, respectively. The softening point of binders also increased. However, the ductility values of the binders decreased. It should be noted that [52] observed maximum and minimum changes in characteristics at 6% PET, after which the changes decreased. Additionally, PET does not negatively impact the aging of binders, making them more resistant to high temperatures and oxidation. The optimal amounts of modifier in [52] were found to be 6% and 8%, where the MBM samples exhibited the best characteristics, including higher resistance to rutting formation.

In [53], it was demonstrated that with an increasing bitumen concentration, the stability value according to Marshall increases up to a certain bitumen content, after which it decreases. Maximum stability was achieved at a level of 5.5%. The optimal binder content across all investigated characteristics of MBM samples in [53] was determined to be 6%.

The microstructures obtained in our study for MBM samples with various percentages of PET particles correspond to the typical microstructures of polymer-modified bituminous binders presented in [29] and are depicted in Table 1.

Since the addition of PET leads to reductions in the needle penetration depth, softening point temperature, and water absorption, it significantly improves the strength and elastic properties of the modified binder, thereby reducing susceptibility to deformation and increasing resistance to rutting and cracking. This phenomenon may be attributed to the swelling of PET particles and their improved compatibility with bitumen.

A data analysis has shown that PET contents at 5% and 8% provide the best properties for modified bitumen and are considered optimal. These findings are consistent with results from other scientific studies available in the literature [47,48,53,54,55]. Increasing the plastic content beyond 8% of PET modifier is not economically viable due to increased production costs of modified bitumen and a decrease in certain performance indicators.

In the present study, comprehensive scientific investigations of bituminous mixtures were conducted, employing a wide variety of modern research methods. Rational and relevant experimental methods for the study of modified bituminous mixtures were selected, enabling a thorough examination of the samples and revealing patterns of structural changes, moisture saturation, and mechanical properties in relation to the percentage of PET modifiers. In other similar works [1,9,11,12,15,16,17,18,20,21,22,56], a significantly smaller number of research methods are employed.

The subsequent study will present the obtained results regarding the influence of PET modifiers on the water resistance and strength characteristics of bituminous mixtures compared to analogous systems, specifically the property changes in bitumen, through blending with other plastics such as high-density polyethylene, low-density polyethylene, polypropylene, polystyrene, ethylene-vinyl acetate copolymer, polyvinyl chloride, and other modifiers. This investigation will highlight both the advantages and disadvantages of modifying bituminous mixtures with plastic waste and will help identify the optimal type of modifier. Additionally, the impact of low temperatures on the operational properties of various bituminous mixtures will be examined, and an analysis of the swelling effect of particles after the moisture saturation of the bituminous mixtures (including the microstructure control of the samples before and after moisture saturation) and its influence on strength characteristics will be conducted.

## 5. Conclusions

The obtained results demonstrate that the introduction of polymer additives positively affects the characteristics of bituminous binders, enhancing their strength, elasticity, aging resistance, and various other properties. Regarding the softening point temperature and tensile strength of the bituminous binder, samples containing 8% mass of polymer waste are optimal. At a 10% PET content, the tensile strength of the modified bitumen mixture sharply declines. Samples with 5% mass and 8% mass polymer contents also exhibit optimal characteristics in terms of the penetration depth and brittle point temperature, with further increases in the polymer content showing only minor improvements in these parameters.

Considering the combination of physico-chemical and operational characteristics, it can be concluded that bituminous binder containing 5% mass and 8% mass of PET waste is the most optimal across all parameters.

The analysis of thermal effects in modified bituminous mixtures revealed that the elasticity of the mixture decreases with the introduction of PET particles exceeding 20%, leading to increased cracking tendencies in the samples. Pure polyethylene terephthalate (PET) without bitumen exhibits a glass transition onset at 82 °C, whereas with bitumen, this onset occurs at 71 °C. A similar trend is observed for melting temperatures, which decrease in the presence of bitumen compared to pure PET.

The fatigue life of the mixture significantly improves following the addition of PET particles into the bitumen mixture, particularly in low-stress conditions, and the differences between samples decrease with increasing stress levels. Thus, the fatigue life of the modified bituminous mixture can be significantly enhanced by incorporating a PET modifier, especially under low loads.

The addition of polyethylene terephthalate (PET) into polymer-modified bituminous binders enhances their water resistance without compromising elasticity while reducing the glass transition temperature, crystallization temperature, and melting temperature of the material. The dependencies of mass growth and rates of moisture absorption were constructed to investigate the water resistance of the samples of the modified bituminous mixture. It was determined that an increase in the percentage of the modifier leads to a decrease in the water mass in the samples.

The innovativeness of the conducted research lies in determining the optimal percentage ratio (5% and 8%) of PET additive in the modified bituminous mixture. Based on the obtained results, developers of bituminous mixtures will be able to produce optimal compositions with the best performance properties, reduce economic costs associated with mixture modification, and improve environmental conditions through the recycling of harmful plastics.

A subsequent study will present the results regarding the influence of PET modifiers on the water resistance and strength characteristics of bituminous mixtures compared to analogous systems, specifically the alteration of bitumen properties through blending with other plastics, such as high-density polyethylene, low-density polyethylene, polypropylene, polystyrene, ethylene-vinyl acetate copolymer, polyvinyl chloride, and other modifiers. This investigation will reveal both the advantages and disadvantages of modifying bituminous mixtures with plastic waste and will aid in identifying the most effective type of modifier.

The conducted research represents the first stage of work upon which subsequent articles will be based. Future investigations will include the following:

− An examination of the effects of low temperatures on the operational properties of various bituminous mixtures;
− An analysis of the impact of modifiers derived from different plastics on the water resistance and strength characteristics of bituminous mixtures to identify the optimal type of modifier;
− An investigation of the swelling effect of particles after the moisture saturation of bituminous mixtures (including the microstructure control of the samples before and after moisture saturation) and its influence on strength characteristics.

The practical and scientific value of this research lies in determining the optimal percentage ratios (5% and 8%) of PET additive in the modified bituminous mixture. The results of this study will be utilized to develop optimal compositions of bituminous mixtures for use in road construction in Kazakhstan.

## Figures and Tables

**Table 1:** The characteristic features of modified bituminous mixture samples based on the analysis of certain surface microstructures.

No	Microstructures of MBM Samples	Characteristic Features of Modified Bituminous Mixture Samples
1	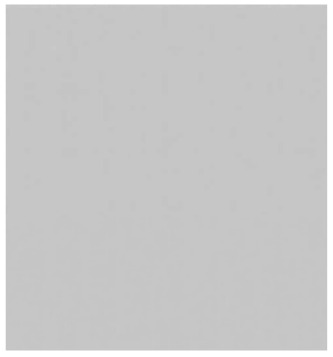	Homogeneous microstructure of MBM sample
2	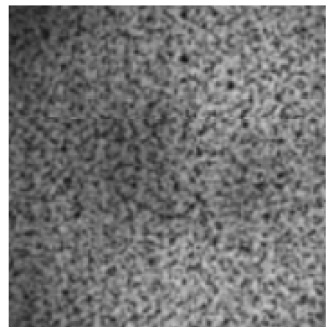	Good polymer distribution with virtually indistinguishable phases
3	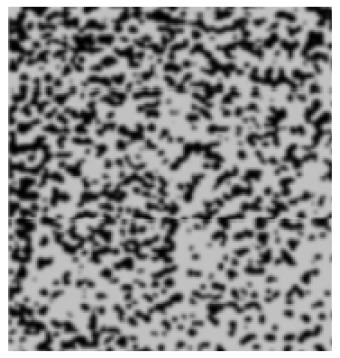	Reduced compatibility between bitumen and polymer (phase inversion observed)
4	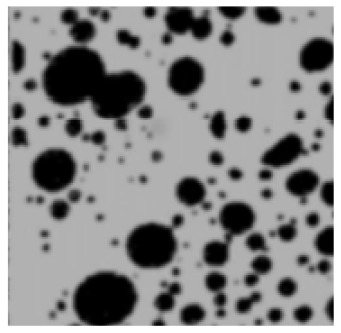	Increase in size of residual asphaltene phase formations
5	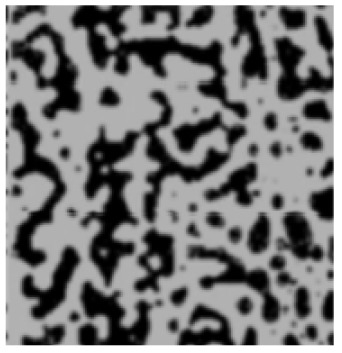	Joint continuous morphology
6	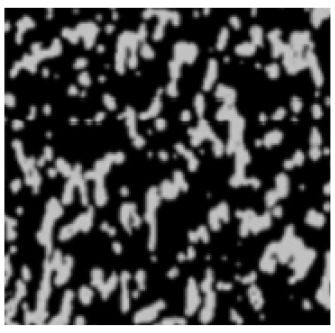	Increase in size of residual asphaltene phase formations
7	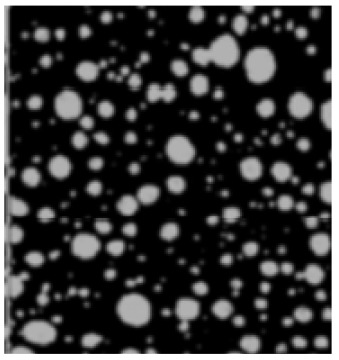	Spherical shape of domains, corresponding to minimal phase boundary

Source: [29].

**Table 2:** Key research methods for modified bituminous mixtures.

No.	Methods and Regulatory Documents	Essence of Methods
1	ASTM D36/D36M-14: Standard test method for softening point of bitumen (ring-and-ball apparatus) [30]	This research method allows for determining the strength of modified bituminous mixture samples and the phase transition temperature from the solid state to the liquid state. It characterizes the thermal stability of modified bituminous mixture samples and their tendency to flow under heating conditions.
2	EN 12593: 2015: Bitumen and bituminous binders—determination of Fraass breaking point [31]	This research method allows for determining the temperature at which cracks appear in samples of modified bituminous mixtures, which can lead to their failure. Thermo-mechanical testing is performed under conditions of repeated static bending of modified bituminous mixture samples at low temperatures.
3	EN 13589: 2014: Bitumen and bituminous binders—determination of tensile properties of bituminous binders by tensile test method [32]	This research method allows for determining the ductility of modified bituminous mixture samples under static tensile loading until failure.
4	EN 1426: 2015: Bitumen and bituminous binders—determination of needle penetration [33]	The degree of penetration is determined as the distance in ten millimeters that a standard needle penetrates vertically into the sample material at a constant temperature. The standard penetration test was conducted at 25 °C over 5 s using the APN-360MG4 penetrometer.

Source: [18].

**Table 3:** Physical and mechanical characteristics of investigated bitumen samples.

No.	Characteristic Name	Temperature, °C	Standard Value	Actual Value
1	Density	25 °C	950–1500 kg/m^2^	1100 kg/m^2^
2	Needle penetration depth	25 °C	from 70 to 100 mm	96 mm
3	Elongation	25 °C	62 cm	54 cm
		0 °C	3.7 cm	3.7 cm
4	Softening point	70 °C	up to 70 °C	68 °C
5	Brittleness	−15 °C	−15 °C	−15 °С

Source: [18].

**Table 4:** Physical and mechanical properties of PET.

No.	Physical and Mechanical Properties of PET	Value
1	Molecular Weight, g/mol	60,000
2	Density, g/cm^3^	138
3	Tensile Strength, MPa	170
4	Elongation at Break, %	50
5	Longitudinal Elastic Modulus, MPa	1700
6	Impact Strength, kJ/m^2^	80
7	Frost Resistance, °C	−50
8	Water Absorption, %	30
9	Decomposition Temperature, °C	345
10	Melting Temperature, °C	245
11	Glass Transition Temperature, °C	70

**Table 5:** Comparative analysis of samples of modified bituminous mixtures and their physico-mechanical properties.

No.	Indicator	Value						
1	Modifier Content, %	0	1	3	5	8	10	12
2	Density, kg/m³	1100	1110	1127	1142	1218	1230	1276
3	Needle Penetration Depth at 25 °C, mm	96	73	71	67	56	53	50
4	Tensile Strength at 25 °C, cm	64	64	61	163	251	134	111
5	Softening Point, °C	72	75	78	85	98	99	100
6	Fracture Temperature, °C	−15	−21	−23	−25	−33	−34	−35
7	Water Absorption, %	18.4	15.9	13.6	12.2	9.8	7.5	6
8	Tensile Strength at Break, MPa	4.1	4.4	6.2	8.5	9.5	7.8	7.5

## Data Availability

The original contributions presented in the study are included in the article, further inquiries can be directed to the corresponding author.

## References

[1] Yadav A., Kumar N., Upadhyay A., Pratibha, Anurag R.K. (2023). Edible packaging from fruit processing waste: A comprehensive review. Food Rev. Int..

[2] Gulyaev A.I., Erasov V.S., Oreshko E.I., Utkin D.A. (2022). Analyzing the destruction of a carbon-fiber-reinforced polymer during the pushout of a multifilamentary cylinder. Polym. Sci. Ser. D.

[3] Oliani W.L., Pusceddu F.H., Parra D.F. (2022). Silver-titanium polymeric nanocomposite non ecotoxic with bactericide activity. Polym. Bull..

[4] Hakuzimana J. (2021). Break free from plastics: Environmental perspectives and evidence from Rwanda. Environ. Ecosyst. Sci..

[5] Liang Y., Tan Q., Song Q., Li J. (2021). An analysis of the plastic waste trade and management in Asia. Waste Manag..

[6] Cheng H., Chen L., McClements D.J., Yang T., Zhang Z., Ren F., Miao M., Tian Y., Jin Z. (2021). Starch-based biodegradable packaging materials: A review of their preparation characterization and diverse applications in the food industry. Trends Food Sci. Technol..

[7] Tian X., Han S., Wang K., Shan T., Li Z., Li S., Wang C. (2022). Waste resource utilization: Spent FCC catalyst-based composite catalyst for waste tire pyrolysis. Fuel.

[8] Moghaddam T.B., Karim M.R., Soltani M. (2013). Utilization of waste plastic bottles in asphalt mixture. J. Eng. Sci. Technol..

[9] Abuaddous M., Taamneh M.M., Rabab’Ah S.R. (2021). The potential use of recycled polyethylene terephthalate (RPET) plastic waste in asphalt binder. Int. J. Pavement Res. Technol..

[10] Xu X., Chen G., Wu Q., Leng Z., Chen X., Zhai Y., Tu Y., Peng C. (2022). Chemical upcycling of waste PET into sustainable asphalt pavement containing recycled concrete aggregates: Insight into moisture-induced damage. Constr. Build. Mater..

[11] Qaidi S., Al-Kamaki Y., Hakeem I., Dulaimi A.F., Özkılıç Y., Sabri M., Sergeev V. (2023). Investigation of the physical-mechanical properties and durability of high-strength concrete with recycled PET as a partial replacement for fine aggregates. Front. Mater..

[12] Jiang X., Titi H., Ma Y., Polaczyk P., Zhang M., Gabrielson J., Bai Y., Huang B. (2022). Evaluating the performance of inverted pavement structure using the accelerated pavement test (APT). Constr. Build. Mater..

[13] Lu D., Jiang X., Tan Z., Yin B., Leng Z., Zhong J. (2023). Enhancing sustainability in pavement Engineering: A-state-of-the-art review of cement asphalt emulsion mixtures. Clean. Mater..

[14] Nizamuddin S., Boom Y.J., Giustozzi F. (2021). Sustainable polymers from recycled waste plastics and their virgin counterparts as bitumen modifiers: A comprehensive review. Polymers.

[15] Jiang X., Gabrielson J., Huang B., Bai Y., Polaczyk P., Zhang M., Hu W., Xiao R. (2022). Evaluation of inverted pavement by structural condition indicators from falling weight deflectometer. Constr. Build. Mater..

[16] Moghaddam T.B., Soltani M., Karim M.R. (2014). Evaluation of permanent deformation characteristics of unmodified and Polyethylene Terephthalate modified asphalt mixtures using dynamic creep test. Mater. Des..

[17] Islam M.M., Shirin M.S., Tonoy T.R., Sweet S.A. (2021). Modification of bitumen properties using waste polymer in context of Bangladesh. Int. J. Min. Proc. Extr. Met..

[18] Chen Z., Leng Z., Jiao Y., Xu F., Lin J., Wang H., Cai J., Zhu L., Zhang Y., Feng N. (2022). Innovative use of industrially produced steel slag powders in asphalt mixture to replace mineral fillers. J. Clean. Prod..

[19] Daly W.H., Collier J.R., Negulescu I.I., Qiu Z., Runkle J. (1995). Determination of Significant Factors Controlling Compatibility of Asphalts with Synthetic Polymers. Technical Report. https://rosap.ntl.bts.gov/view/dot/22244.

[20] Moghaddam T.B., Karim M.R., Syammaun T. (2012). Dynamic properties of stone mastic asphalt mixtures containing waste plastic bottles. Const. Build. Mater..

[21] McNally T. (2011). Polymer Modified Bitumen: Properties and Characterisation.

[22] Makarov D., Ayupov D., Murafa A., Khozin V. (2014). Compatibility studies of mixed thermoplastic rubber with road bitumen. Open. Civ. Eng. J..

[23] Tian Y., Li H., Sun L., Zhang H., Harvey J., Yang J., Yang B., Zuo X. (2021). Laboratory investigation on rheological, chemical and morphological evolution of high content polymer modified bitumen under long-term thermal oxidative aging. Const. Build. Mater..

[24] Plewa A., Belyaev P.S., Andrianov K.A., Zubkov A.F., Frolov V.A. (2016). The effect of modifying additives on the consistency and properties of bitumen binders. Adv. Mater. Technol..

[25] Rossi C.O., Spadafora A., Teltayev B., Izmailova G., Amerbayev Y., Bortolotti V. (2015). Polymer modified bitumen: Rheological properties and structural characterization. Colloids Surf. A Physicochem. Eng. Asp..

[26] Oreshko E.I., Erasov V.S., Lashov O.A., Yakovlev N.O. (2022). Stability study of monolithic and layered plates under compression. Inorg. Mater Appl. Res..

[27] Erasov V.S., Oreshko E.I. (2019). Short-time creep under soft loading and hard loading. Mater. Sci..

[28] Oreshko E.I., Erasov V.S., Lutsenko A.N. (2016). Mathematical modeling of deformation constructional carbon fiber at a bend. Aviat. Mater. Technol..

[29] Polacco G., Filippi S., Merusi F., Stastna G. (2015). A review of the fundamentals of polymer-modified asphalts: Asphalt/polymer interactions and principles of compatibility. Adv. Colloid Interface Sci..

[30] (2020). Standard Test Method for Softening Point of Bitumen (Ring-and-Ball Apparatus).

[31] (2015). Bitumen and Bituminous Binders. Determination of the Fraass Breaking Point.

[32] (2014). Bitumen and Bituminous Binders—Determination of the Tensile Properties of Modified Bitumen by the Force Ductility Method. German Version prEN 13589:2014.

[33] (2015). Bitumen and Bituminous Binders—Determination of Needle Penetration. German version EN 1426:2015.

[34] Gupta S., Patıdar D., Baboo M., Sharma K., Saxena N.S. (2010). Investigation of Al Schottky junction on n-type CdS film deposited on polymer substrate. Front. Optoelectron. China.

[35] Oreshko E.I., Erasov V.S., Jastrebov A.S. (2019). Prediction of strength and deformation characteristics of materials during tension and creep tests. Mater. Sci..

[36] Erasov V.S., Oreshko E.I. (2018). Reasons for dependence of mechanical characteristics of material fracture resistance on sample sizes. Aviat. Mater. Technol..

[37] Ben Zair M., Jakarni F., Muniandy R., Hassim S. (2021). A brief review: Application of recycled polyethylene terephthalate in asphalt pavement reinforcement. Sustainability.

[38] El-Naga I.A., Ragab M. (2019). Benefits of utilization the recycle polyethylene terephthalate waste plastic materials as a modifier to asphalt mixtures. Constr. Build. Mater..

[39] Sojobi A., Nwobodo S.E., Aladegboye O.J. (2016). Recycling of polyethylene terephthalate (PET) plastic bottle wastes in bituminous asphaltic concrete. Cogent. Eng..

[40] Ashoor A., Kareem M.M., Al-Baiati M.N. (2019). Improved asphalt binder using recycle polyethylene terephthalate polymer. IOP Conf. Ser. Mater. Sci. Eng..

[41] Al-Jumaili M.A.H. (2018). Sustainability of asphalt paving materials containing different waste materials. IOP Conf. Ser. Mater. Sci. Eng..

[42] Kakar M.R., Mikhailenko P., Piao Z., Bueno M., Poulikakos L. (2021). Analysis of waste polyethylene (PE) and its by-products in asphalt binder. Constr. Build. Mater..

[43] Ahmad A.F., Razali A.R., Razelan I.S.M., Jalil S.A., Noh M.M., Idris A.A. (2017). Utilization of polyethylene terephthalate (PET) in bituminous mixture for improved performance of roads. IOP Conf. Ser. Mater. Sci. Eng..

[44] Cong L., Peng J., Guo Z., Wang Q. (2017). Evaluation of fatigue cracking in asphalt mixtures based on surface energy. J. Mater. Civ. Eng..

[45] Moghaddam T.B., Soltani M., Karim M.R. (2014). Experimental characterization of rutting performance of Polyethylene Terephthalate modified asphalt mixtures under static and dynamic loads. Constr. Build. Mater..

[46] Choudhary R., Kumar A., Murkute K. (2018). Properties of waste polyethylene terephthalate (PET) modified asphalt mixes: Dependence on PET size, PET content, and mixing process. Period. Polytech. Civ. Eng..

[47] Rahmani E., Dehestani M., Beygi M.H.A., Allahyari H., Nikbin I.M. (2013). On the mechanical properties of concrete containing waste pet particles. Construct. Build. Mater..

[48] Ahmadinia E., Zargar M., Karim M.R., Abdelaziz M., Shafigh P. (2011). Using waste plastic bottles as additive for stone mastic asphalt. Mater. Des..

[49] Poulikakos L.D., Papadaskalopoulou C., Hofko B., Gschösser F., Cannone Falchetto A., Bueno M., Arraigada M., Sousa J., Ruiz R., Petit C. (2017). Harvesting the unexplored potential of European waste materials for road construction. Resour. Conserv. Recycl..

[50] Shiryaev A.O., Obukhov A.G., Vysotskaya M.A., Shekhovtsova S.Y. (2017). Polymer Modifiers of Bitumen Binders.

[51] Ince C.B., Geckil T. (2022). Effects of recycled PET and TEOA on performance characteristics of bitumen. J. Croat. Assoc. Civ. Eng..

[52] Mahrez A., Karim M.R. Rheological evaluation of bituminous binder modified with waste plastic material. Proceedings of the 5th International Symposium on Hydrocarbons & Chemistry (ISHC5).

[53] Prasad A.R., Sowmya N.J. (2015). Bitumen modification with waste plastic and crumb rubber. Int. J. Eng. Res. Technol..

[54] Bary E.M.A., Farag R.K., Ragab A.A., Abdel-Monem R.M., Abo-Shanab Z.L., Saleh A.M.M. (2020). Green asphalt construction with improved stability and dynamic mechanical properties. Polym. Bull..

[55] Hu J., Fang C., Zhou S., Jiao L., Zhang M., Wu D. (2015). Rheological properties of packaging-waste-polyethylene-modified asphalt. J. Vinyl Addit. Technol..

[56] Safonov V.A., Danilova V.N., Ermakov V.V., Vorobyov V.I. (2019). Mercury and methylmercury in surface waters of arid and humid regions, and the role of humic acids in mercury migration. Per. Tchê Quím..

